# An Evidence-Based Practice Developed *in-situ*: Let's Talk About Children and a Consolidation of Its Evidence Base

**DOI:** 10.3389/fpsyt.2022.824241

**Published:** 2022-02-14

**Authors:** Becca Allchin, Tytti Solantaus

**Affiliations:** ^1^Eastern Health, Mental Health Program, Melbourne, VIC, Australia; ^2^School of Rural Health, Monash University, Melbourne, VIC, Australia; ^3^Department of Public Health and Welfare, Finnish Institute for Health and Welfare, Helsinki, Finland

**Keywords:** Evidence-based practice, Let's Talk about Children, family-focused practice, parental mental health, mental health promotion, child wellbeing, prevention in child mental health

## Abstract

**Background:**

Traditional models of evidence-based practice assume knowledge is developed in research settings before being installed in practice settings. The role practice settings can play in enhancing effectiveness and enabling sustainability is not therefore acknowledged. Developing interventions *in-situ* alongside developing their evidence base, provides another pathway to evidence-based practice. One example is Let's Talk about Children (LTC), a brief family-focused intervention that promotes parent, family and child wellbeing. Let's Talk about Children has been developed and adapted to respond to the context into which it has been established, leading to different descriptions reported in its 20 year collection of evidence. Collating the diverse literature on LTC, this paper showcases an evidence-based practice developed *in-situ* in order to guide future innovation.

**Method:**

Using an integrative review, key literature using LTC were identified through electronic databases and snowballing techniques. Constant comparison analysis synthesized the data to develop patterns and themes.

**Findings:**

From the 26 records, three forms of LTC were identified and outcomes related to parents, family and child wellbeing, implementation and sustainability were collated. Consolidated outcomes show overall agreement in effectiveness and acceptability outcomes across different settings and populations. Implementation and sustainability impacts are entwined with the context, and influenced by its development *in-situ*.

**Conclusions:**

The study documents that the *in-situ* model is effective at developing sustainable evidence-based practice. In consolidating the evidence, the review clarified LTC's forms and outcomes, and draws attention to the importance of research on mechanisms of change.

## Introduction

Evidence-based practice emerged in the concept of evidence-based medicine in the 1990's (1) which stressed applying evidence from relevant research to clinical decision making rather than relying on intuition. Evidence-based practice's endorsement led to the rise of interventions created and tested under research conditions that would then be disseminated to practice. This has been described as a ‘pipeline' process; developing *efficacy*–testing if a practice could work under tightly controlled conditions, then *effectiveness*–testing if it does work in less controlled conditions, before *disseminating*–getting the practice to be utilized in service settings (2, 3). Emphasis on each of those phases has shifted, with efficacy dominating the early years of evidence-based practice, moving to effectiveness to build more generalizability and then to dissemination to improve uptake in routine practice (3, 4). More recently, this latter phase has shifted to a focus on implementation, seen as a more active process of equipping services to adopt and sustain such practices (4).

The concept of applying evidence to practice is hard to argue with, however, debate about what constitutes as evidence, and how it is applied has raised questions about the concept and development of evidence-based practices (5–7). The pipeline approach to developing interventions has resulted in interventions that may appear successful but not continue to provide benefits to end-users due to difficulties in implementing or sustaining them in practice (8). Additionally, the 15–20-year process can result in the implemented practice being already outdated by new evidence (4). Hawe (7) indicated that the pipeline process assumes a unidirectional pathway from research to practice, with knowledge developed in research domains before being “installed” into practice domains. Such a unidirectional process of knowledge development does not recognize the role practice settings can have in shaping evidenced-based practices in general, and especially where local-level adaptations may be important for enhancing effectiveness or driving sustainability (7, 9–11).

Another pathway to evidence-based practice has placed a greater value on the practice setting, by developing and adapting interventions *in-situ* while building evidence. One example of this is the Finnish, *Lapset puheeksi*, or in English, Let's Talk about Children (LTC), a family-focused practice with a specific emphasis on the parenting role and the needs of their children (12). The second author developed the first version in 2001, as a component of the Effective Child and Family (ECF) program [*Toimiva lapsi* & *perhe -työ]*, a promotive and preventative approach to child wellbeing which included a suite of tools as documented in Table 1 (12, 20, 21). A large ongoing government-supported nation-wide initiative, the ECF program included training, implementation and research. It aimed to equip health and social services to meet the minimum standards of the Finnish Child Welfare Act to address dependent children's need for care and support (12, 20, 22).

**Table 1:** Effective child and family program's suite of tools.

**ECF suite of tools^**a**^**	**Purpose**	**Details**
Let's Talk about Children Discussion (LT-D)	Map child's life & develop an action plan to promote child's wellbeing	2–3 structured conversations between parent & practitioner. These include an invitation, and two structured conversations using an age-appropriate log and providing parents with the guidebooks (13)
Let's Talk about Children Network meeting (LT-N) also known as Effective Family Network meeting (EFN)	Build a network around the child & family	Parent & practitioner identify people to help facilitate wellbeing of the child i.e., family's own network of supports & services such as child psychiatry, school, housing (14).
Information booklets for parents & young people (12).	Self-guided psychoeducational material	How can I help my children? A guidebook for parents with mental health problems or issues (15) How can I care for my children? A guidebook for parents struggling with drug or alcohol use (16) What's up with our parents? A guidebook for young people whose parents have a mental health problem (17)
Family Talk Intervention (FTI) also known as The Effective Child & Family Intervention (ECFI)/Beardslee Family Intervention, Family Intervention, Preventive Family Intervention (PFI) or Let's Talk Family intervention	Facilitated family conversations by practitioner	A 6–8 session practitioner-led intervention that facilitates conversations between parents and children about the impact of the mental illness on family life (18)
Vertti child and parent group activities^b^	Peer support group program	A 10-week parallel peer support psychoeducation group for children and their parents (19)

*^a^information and links to training can be found at https://mieli.fi/en/development-projects/effective-child-and-family-work*.

*^b^ECF training does not include training in this program*.

LTC served as a control group intervention to a more resource intensive preventative family intervention, ‘Family Talk Intervention' (FTI) in the ECF program (18, 22). LTC was created to fit a health system with limited capacity to provide intensive family treatment for all consumers who were parents (12). So as to be used in adult-focused services, LTC was designed to be delivered by professionals with no experience or training in child development and assessment in the course of their ordinary work (12).

The purpose of LTC is to promote family mental wellbeing while also mitigating and/or preventing mental health issues for both parents and children (12). LTC takes an ecological understanding of child development, resilience and wellbeing that sees the child in the context of their relationships with their environment (23). Central to LTC is engaging parents in the support of their children. It works from the assumption that families are key resources for supporting child wellbeing and that everyday interactions are the stage on which child development plays out (22). Along with research and clinical experience, LTC's development was informed by international interventions for families where a parent has a mental illness including a Dutch mini-intervention and the US-originated FTI (12).

LTC is described as a “low threshold public health intervention” (23) because it is brief, low resource-intensive and has been applied in different settings and with different populations (12, 24). It has been translated, adapted and utilized across a range of countries and cultures including Estonia, Norway Sweden, Greece (25), Japan (26), Australia (27–29) and the USA (30, 31).

Drawing together the evidence for a practice developed *in-situ* can pose unique complications. As it is adapted and developed to fit the practice settings and the population, the way it is described in the literature can vary and its focus audience differ. As a consequence, a clear understanding of the evidence-base can be challenging.

Using LTC as an example, this paper showcases an evidence-based practice developed *in-situ* in order to guide future innovation. The study collates the diverse literature on LTC, to identify its forms and outcomes, and explore the implementation and sustainability impact of developing evidence-base practice *in-situ*. This study used the following questions:

- What was the context of the study (country, population, study type)?
- How was LTC described?
- Was LTC studied alone or with other interventions?
- What outcomes, implementation and sustainability impacts were documented?

## Methods

An integrative review method which permits reviewing qualitative and quantitative literature was used to consolidate what was known about LTC based on the research questions (32).

### Search Strategy and Eligibility Criteria

Key literature on LTC published from 2001-July 2021 was sourced through six health and social databases (Medline, APA PsychArticles, PsychInfo, Embase & Embase plus, Emcare, CINAHL, Scopus) using the search terms of Let^*^ Talk about the Children and Let^*^ Talk about Children. Additional peer-reviewed and gray literature was found through “snowballing techniques” (33) and direct contact with developers and implementers. Given the limited articles published, no exclusion criteria were applied except being published in English and that it met the criteria of documenting outcomes for LTC.

### Screening, Selection, and Data Extraction

**Figure 1:**
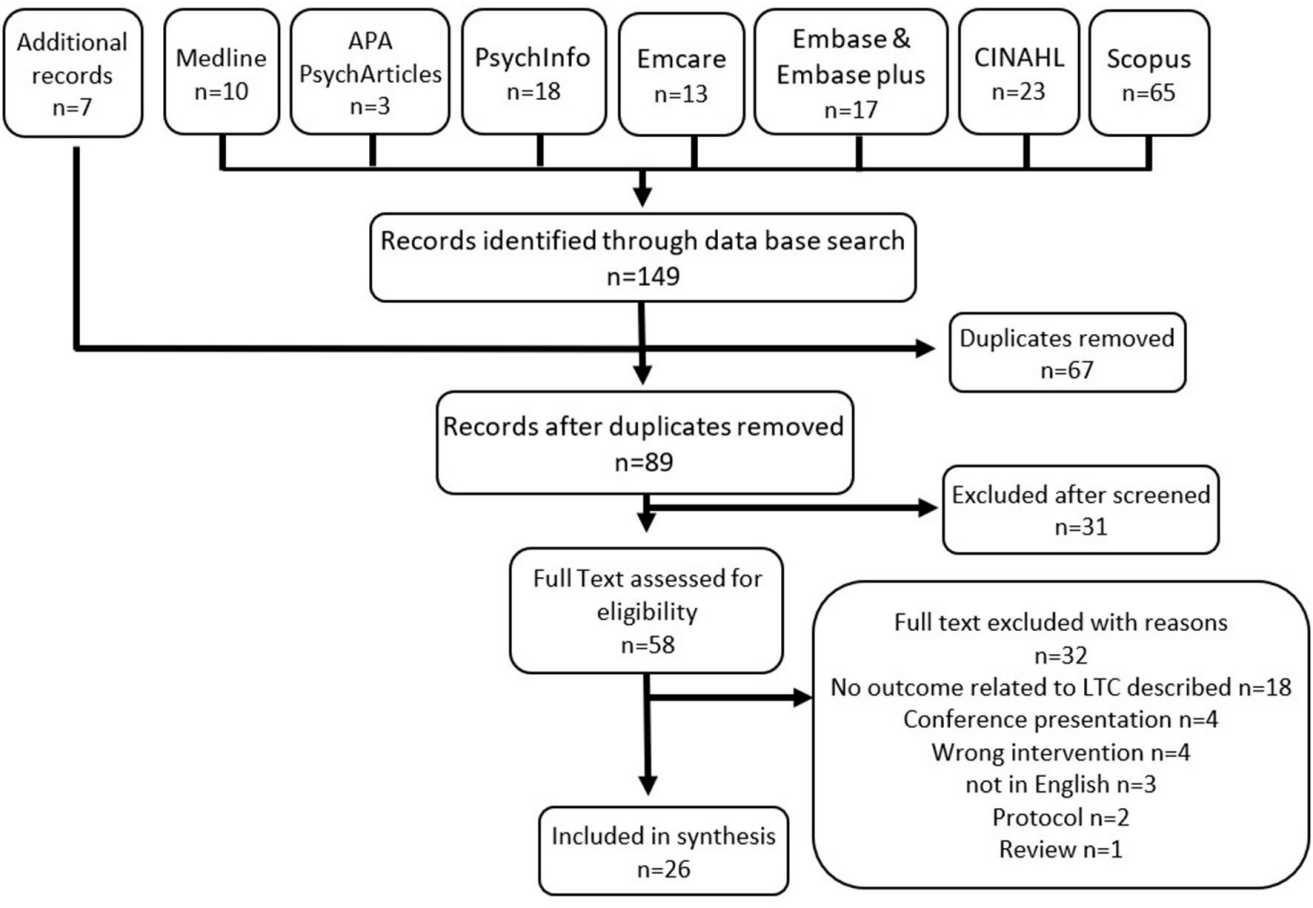
Search and screening.

A total of 149 records were identified via the database search with an additional 7 records via snowballing. After duplicates were removed, 89 records were screened at title and abstract removing an additional 31 records. The remaining records' full text were then assessed for documenting outcomes for LTC, resulting in 26 records included in review (see Figure 1).

### Analysis

Data was extracted by the primary author and entered into a matrix according to review questions. Constant comparison was used to group extracted data into systematic categories to enable data synthesis through the identification of patterns and themes consistent with integrative review methods (32).

## Findings

### Records

The study identified 26 records documenting outcomes for LTC; five of randomized control trials (RCT), three Quantitative papers, five Qualitative papers, seven of mixed method studies and six descriptive and commentary records (see Supplementary Materials 1, 2). The records highlight that the emerging evidence-base of LTC is derived from a set of discrete research endeavors in diverse settings beginning in Finland and now including Greece, Japan, Australia and USA. The variety of settings included adult mental health settings both clinical and Non-government, general hospital psychiatry, child and family services and universal settings (12, 20, 21, 25, 27, 34–36). The range of populations studied included families where a parent has depression, bipolar disorder, life threatening cancer, schizophrenia, schizoaffective disorder, borderline personality disorder, anxiety, Post-traumatic stress disorder, gambling and other co-occurring issues. The early studies were of the version of LTC developed for the RCT (LT-1) and later have been on the manualised intervention of two or three sessions (LT-D) designed for either treatment or universal settings. In some studies, LTC has been included as part of a suite of interventions (12, 20, 25, 35, 37). RCT's on LTC have compared it to a more intensive family intervention (20, 22, 25, 37) as well as to usual practice (36).

The research endeavors in regards to LTC have included a focus on its effectiveness, safety and acceptability in its different forms, in different settings and in different populations (20, 25–27, 38). There is also documentation of its implementation (12, 21, 29, 30, 34, 39) and on its sustainability (21, 40–42).

### Different Forms of LTC

The records document LTC as evolving to fit its context. While the controlled adaptations to LTC resulted in changes in the way it was described in the literature, its different forms are recognized as developments of the same intervention as noted in Table 2.

**Table 2:** Descriptions of the versions of LTC.

**Let's Talk about Children Versions**	**Details**
Let's Talk about Children Discussion-One (LT-1)	Early version of LTC used in the RCT with a conversation guide but without the structured log. Documented as conversations with parents about their children and providing parents with the guidebooks taking between one 15 min or two 45 min sessions. All practitioners, however, used more than 15 min (20) with 75% using one full session and 24% using two sessions (22).
Let's Talk about Children Discussion (LT-D)	Structured version of LTC using a series of 2–3 structured conversations including an introduction invitation and set of two discussions (LT-D) and providing parents with the guidebooks (13, 21, 23, 26). Discussion 1 uses an age-appropriate structured log to assist the parent to map the strengths and vulnerabilities within the everyday encounters and routines in the child's life (23). Discussion 2 builds on the previous discussion exploring how the parents can promote the child's wellbeing through building resilience in the systems around the child. Utilized in two different settings: 1. Child development & education. Early childhood, primary schools & high schools each have own log. 2. Service settings including both in treatment or care settings (i.e., psychiatric services, palliative care units, consultation psychiatry, child protection) and in promotive settings (i.e., maternal child health, community health). Six age-appropriate logs.
Let's Talk about Children Network meeting (LT-N) also known as Effective Family Network meeting (EFN)	An extension to LT-1 and LT-D that facilitates linking the child and family to support by building a network around the child. Used after LT-1 and LT-D as required, the parent identifies people including the family's own network of supports, schools, as well as services such as child psychiatry, housing etc. that may be able to help facilitate the wellbeing of the child (12, 14, 43). This became the second step of the two-step model of LTC.
Let's Talk about Children Service Model (LT-SM)	Use of LTC for collective impact through connecting systems across whole regions. Regional implementation strategy starts with community engagement and includes establishing a regional senior management group to enable service coordination and collaboration, as well as local management groups to oversee local implementation (35). Includes the two-step model of LTC: the parent and worker first use LT-D to chart the child's everyday life and develop an action plan to enhance strengths and support vulnerability. If a second step is needed, the LT-N is used to broaden the network of support for the child and family (35).

Initially, LTC (LT-1) was described as a conversation with parents about their children, and included the provision of guidebooks (15–17) and development of an action plan to address the strengths and vulnerabilities identified in the discussion (12, 20). LTC Network meeting (LT-N), was developed to further address the strengths and vulnerabilities through linking the child and family to support (12, 14, 21). While the LT-N was recommended, at first it was not officially part of LTC.

After the data collection for the ECF RCT ended in 2006, LTC was described as a series of structured conversations including an introduction invitation and a set of two discussions which used a structured log and provided parents with guidebooks (LT-D) (13, 21, 26). The structured log was developed at the request of adult mental health practitioners who, with no training in child mental health, needed more detailed support for conversations about children's strengths, vulnerability and need for further support. Systematically mapping the child's life, it provided a comprehensive picture of the child and family's life and wellbeing.

Subsequently, LTC was adapted to a Finnish public health intervention delivered to the general population without an underlying risk or problem. The motto being “Every child is worth a discussion” (mieli.fi/letstalk). This incorporated a whole-of-region approach with education settings and services working together as part of the national strategy (35, 44). New versions of the log were developed to facilitate the parent, teacher and child (as appropriate) to jointly map the child's life with the aim of creating concrete support for the everyday life of the child also at school and in daycare. LT-N was incorporated into LTC making it a two-step intervention with LT-D, with municipalities made responsible to organize relevant services and support people to come together for the network meeting (35). This LTC approach, called the Let's Talk about Children Service Model (LT-SM), facilitates systematic promotion of child wellbeing and development in universal settings (35).

### Evidence Base

#### Summary of Evidence Base

The first RCT on LTC, carried out in Finland, was based on LT-1 (20–22, 37), with the rest of the studies using the structured LT-D with or without LT-N. LT-N was used in the first RCT but was not officially part of LTC, while in the Greek RCT it was.

As the outcomes of LTC's different versions are in agreement, the following documentation does not differentiate by version. LTC has been found to be acceptable for parents (20, 26, 27) and for mental health practitioners (13, 25, 29, 38). The records make connections between parent, family and child wellbeing outcomes, which are explored in detail below. Additionally, implementation and sustainability outcomes and impacts have been documented both in focused studies (21, 40–42) and from the context of other studies.

##### Parent and Family Outcomes

Studies on LTC document improvements in parents' mental health and wellbeing, in their parenting skills and confidence, and in their relationship with their children. Mental health and wellbeing improvements included decreased anxiety and depression (20, 25–27, 36), increased motivation for mental health treatment (20, 26), improvements in their own social support (25) and a greater future orientation with increased confidence in the child's and family's future (20, 26).

Shifts in parenting included improved confidence in parenting and greater self-acceptance (20, 22, 26) with more parenting ideas (20) and a decrease in parenting stress (27). Parents also reported less guilt, shame and worries about their children (20, 22, 26). Improved parent-child connection was documented through an increased understanding of their children (20, 25). The family outcomes are in line with the parent outcomes, with improved family connection and communication, confidence to talk about mental illness (20, 27), leading to mutual understanding in the family (20, 25, 27).

##### Child Outcomes

LTC studies document a decrease in negative outcomes and an increase in promotive factors for children. Improvements included a decrease in child depression (25), anxiety and behavioral problems (22, 25). Increases were documented for the prosocial behavior needed to solve interpersonal conflicts and promote relationships (22, 25), as well as their subjectively perceived social support and health-related quality of life (25). An increase was also seen in children's positive and functional thinking, which was associated with improvement in children's symptoms of depression and anxiety (37). In two studies, these outcomes were seen later than the parent and family improvements, at 10–18 months after LTC was delivered (22, 37).

Child outcomes were significantly associated with improvements in family functioning (25). Child prosocial behavior, emotional/behavioral problems, anxiety and health related quality of life were also associated with improvements in parenting and the parent's social support (22, 25). The linking of child outcomes to shifts in family processes highlighted LTC as a preventative and promotive intervention for child mental health for families where parental anxiety and depression is present (22, 25, 37). The study by Niemelä et al. (35) documents a significant reduction in children being referred to child protection in the region in which LT-SM was implemented.

##### Implementation and Sustainability Impacts

The records document different contexts impacting implementation and sustainability of LTC. These include diverse approaches to building workforce capacity, adaptations made or required, and commonalities of organizational capacity to support practice.

LTC was developed and implemented to meet a need to develop adult mental health practice that satisfied Finnish minimum standards in welfare and health care legislation (12, 20, 21). Consequently, its implementation in Finland was embedded within a broader government initiative to incorporate promotive and preventative approaches to child wellbeing. LTC was documented as being feasible for use in Finnish general psychiatry and adult mental health settings (13, 20). The ECF approaches, including LTC, were recorded as being in use in two thirds of the health districts in Finland after 5 years and endorsed in national recommendations for Finnish health services (12, 13, 36).

Records note implementation outside Finland as piloting or trialing a cost-effective evidence-based practice and/or testing its applicability to the different cultures or populations (26, 27). In Australia, implementation is documented as part of localized pilots and trials of LTC in adult focused mental health settings in the context of national government supported online training and resource development (38, 45). Time constraints, high caseloads and tension between child protection concerns and the therapeutic relationship are noted as challenges for the fit of family-focused practice including LTC, in clinical mental health services (29, 38, 39). In Greece, implementation was part of a multiphase government-funded 3-year child mental health promotion program that first tested the ECF's fit to the context within the RCT, finding it feasible for use and family culture in Greece. It was then scaled up to 90 mental health services where 529 families received an intervention and a majority of practitioners chose LTC (25). Implementation in Japan is recorded as testing LTC's fit to context, finding it safe and feasible to be used in parents with mood disorders in Japanese culture (26). In the USA, implementation was in the context of a statewide initiative incorporating a research-service collaboration to adapt LTC to their service delivery context (30). The brevity of LTC was identified as promoting its ability to build to scale in public health in Finland (13, 20, 21, 36) and in Greece (25).

To enable LTC to fit these cultures and populations, self-help booklets and the log were translated (25, 26, 38, 45), and handouts were tailored for different settings (cancer, gambling) to guide discussions with children (13, 34). The adapted material was documented as acceptable to parents in Australia and Japan (26, 45).

Different approaches were used for building workforce capacity to deliver LTC. Where implementation was embedded within broader shifts, such as in Finland, building workforce capacity included an initial broad awareness-raising process prior to the method training. Regional campaigns aimed at the public and professionals in health, social services and education sectors, ran open seminars and media coverage. These built awareness on family and child experiences of parental mental disorder, the importance of prevention and promotion in child and family mental health and the basics of preventive interventions (12). The subsequent method training in the new approaches included training and supporting master trainers from within organizations and the provision of practice supervision (12, 13, 21). Training infrastructure for LTC's sustainability in Finland is presumed from documentation of master trainers training others, a pool of trainers, large numbers of trained practitioners (12, 21, 35) and its use in routine practice with families affected by parental cancer (13, 36).

Where implementation was piloting or trialing a cost-effective evidence-based practice, such as Australia and Japan, specific LTC method training is documented as the focus of workforce capacity building. In Australia, this took a variety of approaches; a train the trainer model (27, 46), online training modules only (38) or online with face-to-face training (29). The studies of the latter two, identified a need for support to apply the training to practice and suggested incorporating opportunity for practice into training, observing others' use LTC and Post-training follow-up (29, 38).

In the USA, where implementation was embedded within an adaptation process, a comprehensive change process using a learning collaborative was documented that incorporated in-person training, virtual hubs, coaching and debriefing (30). While costly, this approach was noted to have multifaceted impacts to support implementation (30).

Overall, the training of LTC was identified as effective for increasing practitioners' skill and knowledge about the impact of mental illness on parents, children and families (21, 30, 38, 46) and on supporting families (21, 29, 38). Change in practice is noted with parents reporting having ongoing discussions about family and children after delivery of LT-1 (20) and improvements in practitioners' ability to gauge a parent's understanding of their children, reflect on the impacts on children and work together with the parent to address impacts and provide resources/referrals where necessary (29, 36, 38). Practitioners indicated that using the practice increased their enjoyment and motivation at work (21). All Finnish trained practitioners were noted as using the suite of ECF interventions, implying the use of LTC (21). Documentation specifically of practitioner's delivery of LTC is only documented in one Australian record which noted over half offering and less than half delivering it (41). Adaptations to delivery were also documented, with practitioners delivering it in less sessions or without the structured log to enable LTC to fit everyday practice (29, 41). Such adaptations were not accompanied with monitoring parent, child and family outcomes and core mechanisms of change for LTC are not clearly articulated.

Common organizational capacities important for implementing and sustaining LTC are identified in the records. Organizational ownership with multiple levels of leadership and internal implementers is noted as vital for sustainability (35, 39, 40, 42). Senior leadership in particular was identified as giving or needed to give authority and vision (34, 35, 40, 42) while other leadership was important for integrating into practitioners' everyday work (39, 42). Infrastructure identified as supporting implementation and LTC's continued use included training structures, data collection and feedback systems on parent numbers, training gaps and practitioners use, and integration into committee structures and policy (40, 42). Municipal cross-sector collaboration with multilevel implementation support and regular data collection was seen as important for LT-SM sustained use (35).

## Discussion

We studied the consolidated evidence of LTC as an example of an evidence-based intervention developed *in-situ*, focusing on parent and family, child and implementation and sustainability outcomes. It demonstrates the relevance of this approach to developing evidence-based interventions.

Development *in-situ* means that intervention development is influenced by context and knowledge from evolving experience over time (9). This contrasts to evidence-based practices developed via the pipeline approach which are often understood as finished by the time of implementing into practice settings and universally applicable via an implementation process (7). While adaptations can be seen as threats to fidelity and the lack of sustained practice as an implementation issue, development *in-situ* allows the practice context to influence the shaping of the intervention (47).

Our study demonstrates how intervention development *in-situ* enabled a rapid response to an acknowledged problem, rather than waiting for a fully-developed intervention with a research base. LTC's initial practice was able to immediately address a known need while continuing to evolve, based on knowledge of the practice setting and the needs and experiences of practitioners and family members.

The current review of LTC highlights that developing an intervention to fit the setting while simultaneously developing its evidence-base may also be advantageous for building interventions that can be sustained in real world settings. The alignment between the setting and LTC, vital for sustainability (8), can be seen in this study with increased structure built within the intervention and organizational support as it evolved. Building interventions *in-situ* brings the work on effectiveness and implementation together. Under these circumstances, it is less likely that an intervention that does not fit the practice setting could be deemed effective and the suitability of an intervention is measured in the light of adjustments made within the organization. The documentation of the spread and sustainability of LTC within Finland (12, 21, 35, 36) suggests this approach is a useful pathway to evidence-based practices that fit settings and can be sustained.

A challenge for developing evidence-based interventions *in-situ*, however, is the ability to consolidate the evidence base and draw together a clear understanding of the practice. As seen in this study, the intervention's description shifted as it was adapted to context, culture and population, and outcomes were published in different fields over many years. The three forms of LTC identified in this review are thus consistent with a practice shaped by the setting, with an evolving body of evidence. Regardless of LTC version, the studies document similar outcomes for children, parents and families. The referenced studies in this review, however, lack detailed descriptions of LTC's adjustments and analysis for subsets of families, limiting clear understanding for who it is and is not a good fit for. This remains a task for future studies. Collectively, the evidence also draws attention to possible core mechanism of change for LTC. Having these clearly articulated could promote adaptations that result in the same expected outcomes and provide guidance for its implementation and evaluation.

The example of LTC invites different ways to consider evidence-based practice. Rather than the action of an intervention being defined and manualized, the evidence-base for the core mechanisms of change could be clearly articulated, to enable practices to be fitted to settings. The focus shifts then from fidelity of a manualized intervention, to measuring how the core mechanisms are enacted within practice. As seen for LTC, it is uncommon, however, for these core mechanisms of evidence-based practices to be articulated and have measures identified (48), or for a practice logic or underlying theory to be incorporated into manuals. These will be important to enable consistent evaluation that can build a body of evidence as it is adapted.

## Conclusion

This study set out to showcase an evidence-based practice developed *in-situ* through an integrative review of literature on LTC. In consolidating the evidence, the review clarified how the three forms of LTC reported in the literature, document similar outcomes for children, parents and families, and provide a window into its spread and sustainability. The results suggest that intervention development influenced by the practice context provides benefits for implementation and does not compromise the evidence-base. Intervention development *in-situ* is a relevant developmental pathway for evidence-based practices. Clear articulation of the core mechanisms of change is important for consistent evaluation and reporting the adjustments made in the intervention in different settings will support future *in-situ* evidence-based practice development.

## Data Availability Statement

The original contributions presented in the study are included in the article/Supplementary Materials, further inquiries can be directed to the corresponding author.

## Author Contributions

BA designed and led the data collection and early analysis in consultation with TS. Both authors analyzed the data, contributed and edited the manuscript, and approved the final manuscript.

## Conflict of Interest

The authors declare that the research was conducted in the absence of any commercial or financial relationships that could be construed as a potential conflict of interest.

## Publisher's Note

All claims expressed in this article are solely those of the authors and do not necessarily represent those of their affiliated organizations, or those of the publisher, the editors and the reviewers. Any product that may be evaluated in this article, or claim that may be made by its manufacturer, is not guaranteed or endorsed by the publisher.
