# Supplementary material for: An Evidence-Based Practice Developed in-situ: Let's Talk About Children and a Consolidation of Its Evidence Base
Source: Front Psychiatry. 2022 Feb 14;13:824241. doi: 10.3389/fpsyt.2022.824241 (PMC8882815; doi:10.3389/fpsyt.2022.824241)
Supplement: Supplementary file 1 [file Table_1.pdf]

Supplementary Material 1: Literature reviewed

Author, year, title, journal	LCT type	Paper type	Area	Study population	Country
Toikka S, Solantaus T. (2006) The Effective Family Programme II: clinicians' experiences of training in promotive and preventative child mental health methods. Int J Ment Health Promot. 2006;8(4):4-10 https://doi.org/10.1080/14623730.2006.9721746	LT-1 in ECF	Quantitative descriptive	Adult psychiatry	Pioneer trainers remaining in adult psychiatry (n=36; 30 questionnaire returned).	Finland
Solantaus T, Toikka S. (2006) The Effective Family Programme: preventative services for the children of mentally ill parents in Finland. Int J Ment Health Promot. 2006;8(3):37Y44. https://doi.org/10.1080/14623730.2006.9721744	LT-D & LT-N in ECF	Descriptive	Adult psychiatry		Finland
Solantaus T, Toikka S, Alasuutari M, Beardslee WR, Paavonen J. (2009) Safety, feasibility and family experiences of preventive interventions for children and families with parental depression. Int J Ment Health Promot. 2009;11(4):15Y24. https://doi.org/10.1080/14623730.2009.9721796	LT-1 in ECF	RCT comparing FTI and LT-1 within ECF - safety feasibility	Adult psychiatry	Parents with mood disorders + child 8-16yrs (n=119; Pre 60 FTI 59 LT. Post 45 FTI and 45 LT).	Finland
Solantaus, T., & Puras, D. (2010). Caring for children of parents with mental health problems—a venture into historical and cultural processes in Europe. *International Journal of Mental Health Promotion*, *12*(4), 27-36. https://doi.org/10.1080/14623730.2010.9721823	ECF	Descriptive	Adult psychiatry		Europe
Niemelä, M., Väisänen, L., Marshall, C., Hakko, H., & Räsänen, S. (2010). The Experiences of Mental Health Professionals Using Structured Family-Centered Interventions to Support Children of Cancer Patients. Cancer Nursing, 33(6), E18-E27. https://doi.org/10.1097/NCC.0b013e3181ddfcb5	LT-D & LT-N in ECF	Qualitative	Cancer	Clinicians (n=7)	Finland
Solantaus, T., Paavonen, J. E., Toikka, S., & Punamäki, R.-L. (2010). Preventive interventions in families with parental depression: children's psychosocial symptoms and prosocial behaviour. European Child and Adolescent Psychiatry, 19(12), 883-892. https://doi.org/10.1007/s00787-010-0135-3	LT-1 in ECF	RCT effectiveness of FTI & LT-1	Adult psychiatry	Families (Parents with mood disorders with children 8-16yrs; n=119 83 complete data set)	Finland
Beardslee, W. R., Solantaus, T., Morgan, B. S., Gladstone, T. R., & Kowalenko, N. M. (2012) Preventive interventions for children of parents with depression: international perspectives. The Medical journal of Australia, 199(3 Suppl), S23-S25. https://doi.org/10.5694/mja11.11289	ECF	Descriptive	Adult psychiatry		Finland

Author, year, title, journal	LCT type	Paper type	Area	Study population	Country
Niemelä, M., Repo, J., Wahlberg, K.-E., Hakko, H., & Räsänen, S. (2012). Pilot Evaluation of the Impact of Structured Child-Centered Interventions on Psychiatric Symptom Profile of Parents with Serious Somatic Illness: Struggle for Life Trial. Journal of Psychosocial Oncology, 30(3), 316-330. https://doi.org/10.1080/07347332.2012.664258	LT-D & LT-N	RCT LT-D, FTI and Control	Cancer	Parents with cancer with child 8-17yrs (n=19; baseline+ 4 months)	Finland
Punamäki, R.-L., Paavonen, J., Toikka, S., & Solantaus, T. (2013). Effectiveness of preventive family intervention in improving cognitive attributions among children of depressed parents: a randomized study. Journal of family psychology: JFP : journal of the Division of Family Psychology of the American Psychological Association (Division 43), 27(4), 683. https://doi.org/10.1037/a0033466	LT-1 in ECF	RCT of FTI and LT Baseline, f/u 4, 10 and 18months. This one baseline, 10 and 18 months	Adult Psychiatry	Families (Parents with mood disorders with children 8-16yrs; baseline n=109, 53 FTI, 56 LT). 145 children (76 FTI, 69 LT)	Finland
Tchernegovski, P., Reupert, A., & Maybery, D. (2015). Let's Talk about Children: A pilot evaluation of an e-learning resource for mental health clinicians. Clinical Psychologist, 19(1), 49-58. https://doi.org/10.1111/cp.12050	LTD - Australian	Sequential Mixed Methods - effectiveness of e-learning resource	Adult & Child psychiatry	Practitioners (n=21)	Australia
Solantaus, T., Reupert, A. E., & Maybery, D. J. (2015). Working with parents who have a psychiatric disorder In A. E. Reupert, D. J. Maybery, J. Nicholson, M. Gopfert, & M. V. Seeman (Eds.), Parental psychiatric disorder: distressed parents and their families (3rd ed., pp. 238-247). New York Cambridge University Press.	LT-D & LT-N	Descriptive Book Chapter	Adult Psychiatry		Finland
Bouverie Centre (2015) Project Report - Mental Health Beacon: Implementing family inclusive practices in Victorian Mental Health Services https://www.bouverie.org.au/news/mental_health_beacon_project_report_march_2015	LT-D - Australian with SSFC	Mixed method evaluation with controlled trial.	Adult psychiatry	Parents with mental illness (n=37) Practitioners (n=69) Managers/Champions	Australia
Niemelä, M., Marshall, C. A., Kroll, T., Curran, M., Koerner, S. S., Räsänen, S., & García, F. (2016). Family-Focused Preventive Interventions with Cancer Cosurvivors: A Call to Action. American Journal of Public Health, 106(8), 1381-1387. https://doi.org/10.3389/fpsyt.2019.00064	LT-D & LT-N	Descriptive	Cancer		International - USA & Finland
Cooper, V., & Reupert, A. (2017). “Let’s Talk About Children” resource: A parallel mixed method evaluation. Social Work in Mental Health, 15(1), 47-65. https://doi.org/10.1080/15332985.2016.1170090	LTD - Australian	Mixed methods - feasibility of psychoeducational resource	Adult Mental Health	Parents with mental illness (n=19)	Australia

Author, year, title, journal	LCT type	Paper type	Area	Study population	Country
von Doussa, H., Sundbery, J., Cuff, R., Jones, S., & Goodyear, M. (2017). 'Let's Talk About Children': Investigating the Use of a Family-focused Intervention in the Gambling Support Services Sector. Australian and New Zealand Journal of Family Therapy, 38(3), 482-495. https://doi.org/10.1002/anzf.1233	LT -D Adapted for gambling	Qualitative	Gambling	Practitioners (n=9)	Australia
Solantaus, T. (2017). Commentary: 'Let's Talk about Children': Investigating the Use of a Family-focused Intervention in the Gambling Support Services Sector. Australian and New Zealand Journal of Family Therapy, 38(3), 496-497. https://doi.org/10.1002/anzf.1239	LT-D & LT-N	Commentary /Descriptive	Gambling		Australia/ Finland
Maybery, D. J., Goodyear, M. J., Reupert, A. E., Sheen, J., Cann, W., O'Hanlon, B., & Cuff, R. (2019). A mixed method evaluation of an intervention for parents with mental illness. Clinical Child Psychology and Psychiatry, 1359104518822676. https://doi.org/10.1177%2F1359104518822676	LT-D - Australian with SSFC	Mixed method	Mental Illness	Parents (n=40)	Australia
Niemelä, M., Kallunki, H., Jokinen, J., Räsänen, S., Ala-Aho, B., Hakko, H., Ristikari T and Solantaus, T. (2019). Collective Impact on Prevention: Let's Talk About Children Service Model and Decrease in Referrals to Child Protection Services. Frontiers in Psychiatry, 10(64). https://doi.org/10.3389/fpsyt.2019.00064	LT- SM	Brief Quantitative Research Report	Community	Region	Finland
Ueno, R., Osada, H., Solantaus, T., Murakoshi, A., & Inoue, T. (2019). Safety, Feasibility, Fidelity, and Perceived Benefits of an Intervention for Parents with Mood Disorders and Their Children — "Let's Talk About Children" in Japan. Journal of Family Psychotherapy, 1-20. https://doi.org/10.1080/08975353.2019.1678092	LT-D -Japan	Pilot Mixed method	Adult Psychiatry	Parents with mood disorders with children 8-16yrs (n=9)	Japan
Karibi H. & Arblaster K. (2019) "Clinician experiences of "Let's Talk about Children" training and implementation to support families affected by parental mental illness", The Journal of Mental Health Training, Education and Practice, Vol. 14 No. 4, pp. 201-211. https://doi.org/10.1108/JMHTEP-08-2018-0044	LT-D -Australian	Qualitative	Adult Mental Health	AMHS practitioners (n= 10)	Australia
Allchin, B, Goodyear, M, O'Hanlon, B, Weimand, BM. (2020), Leadership perspectives on key elements influencing implementing a family-focused intervention in mental health services. J Psychiatr Ment Health Nurs. 2020; 27: 616– 627. https://doi.org/10.1111/jpm.12615	LT-D -Australian	Qualitative	Adult Mental Health	Service managers and implementation leads (n=16)	Australia

Author, year, title, journal	LCT type	Paper type	Area	Study population	Country
Allchin, B., O'Hanlon, B., Weimand, B.M., Boyer, F., Cripps, G., Gill, L., Paisley, B., Pietsch, S., Wynne, B., and Goodyear, M. (2020). An explanatory model of factors enabling sustainability of let's talk in an adult mental health service: a participatory case study. Int J Ment Health Syst 14, 48 (2020). https://doi.org/10.1186/s13033-020-00380-9	LT-D -Australian	Qualitative - Participatory case study	Adult Mental Health	AMH service (n=1)	Australia
Allchin, B., O'Hanlon, B., Weimand, B.M. and Goodyear, M. (2020), Practitioners' application of Let's Talk about Children intervention in adult mental health services. Int J Mental Health Nurs, 29: 899-907. https://doi.org/10.1111/inm.12724	LT-D -Australian	Quantitative	Adult Mental Health	Practitioners (n=73) from 8 AMHS	Australia
Allchin, B., Weimand, B.M., O'Hanlon, B. and Goodyear, M. (2020) Continued capacity: Factors of importance for organizations to support continued Let's Talk practice – a mixed-methods study. Int. J. Mental Health Nurs., 29: 1131-1143. https://doi.org/10.1111/inm.12754	LT-D -Australian	Sequential Mixed methods	Adult Mental Health	AMHS (n=8)	Australia
Giannakopoulos G., Solantaus T., Tzavara C. and Kolaitis G (2021) Mental health promotion and prevention interventions in families with parental depression: A randomized controlled trial. Journal of Affective Disorders, 278, 114-121. https://doi.org/10.1016/j.jad.2020.09.070	LT -D	RCT LT-D, FTI and Control	Cross sector	Families (n=62) including a child and parent with depression. LTC (n-32) FTI (n=30)	Greece
Nicholson, J.; English, K.; Heyman, M. (2021) The ParentingWell Learning Collaborative Feasibility Study: Training Adult Mental Health Service Practitioners in a Family-Focused Practice Approach. Community Ment Health J (2021). https://doi.org/10.1007/s10597-021-00818-5	ParentingWell - USA adaptation	Exploratory design and Mixed methods	Adult Mental Health	Practitioners (n=30) from 5 Agencies	USA
