# Supplementary material for: An Evidence-Based Practice Developed in-situ: Let's Talk About Children and a Consolidation of Its Evidence Base
Source: Front Psychiatry. 2022 Feb 14;13:824241. doi: 10.3389/fpsyt.2022.824241 (PMC8882815; doi:10.3389/fpsyt.2022.824241)
Supplement: Supplementary file 2 [file Table_2.pdf]

Supplementary Material 2: Measures and Outcomes

Author	Year	Measures	implementation outcomes	practitioner outcomes	parent outcomes	child outcomes	family outcomes
Toikka S, Solantaus T.	2006	**Practitioners:** Structured Questionnaire	Y	Y			Y
Solantaus T, Toikka S.	2006		Y				
Solantaus T, Toikka S, Alasuutari M, Beardslee WR, Paavonen J.	2009	**Parent**: BDI- II, Questionnaire. **Child:** (FTI only): CDI; Questionnaire	Y		Y	N/A	Y
Solantaus, T., & Puras, D.	2010		Y				
Niemelä, M., Väisänen, L., Marshall, C., Hakko, H., & Räsänen, S.	2010	**Practitioners**: Narrative interviews	Y	Y			
Solantaus, T., Paavonen, J. E., Toikka, S., & Punamäki, R.-L.	2010	**Parents:** BDI; STAI **Child:** SDQ-4 problem scales & prosocial behaviour Ax mother and fathers separately; SCARED. **Practitioners:** Logbook. **4 time periods:** Baseline, 4 10 and 18 month follow up	Y			Y	
Beardslee, W. R., Solantaus, T., Morgan, B. S., Gladstone, T. R., & Kowalenko, N. M.	2012		Y				
Niemelä, M., Repo, J., Wahlberg, K.-E., Hakko, H., & Räsänen, S.	2012	**Child:** CDI; SDQ. **Parents & Children:** SCL-90; CDI; PACS; SOC; IAS; Questionnaire family demographics and child risk and protective factors **2-time points:** baseline, 4-month post-intervention			Y		
Punamäki, R.-L., Paavonen, J., Toikka, S., & Solantaus, T.	2013	**Child & Parent:** CASQ-R; CDI; Emotional Problem Scale of SDQ. **3-time points:** baseline, 10- and 18-months post-intervention	Y			Y	
Tchernegovski, P., Reupert, A., & Maybery, D.	2015	**Practitioners:** Telephone interviews. FFWQ (shorter version of FFMHPQ - 7 of these subscales)	Y	Y			
Solantaus, T., Reupert, A. E., & Maybery, D. J.	2015		Y				

Author	Year	Measures	implementation outcomes	practitioner outcomes	parent outcomes	child outcomes	family outcomes
Bouverie Centre	2015	**Practitioners:** FFMHQ; Logbook, FIS; n=43. **Manager and Champions:** Interviews. **Parents:** Questionnaire therapeutic change (n=23)	Y	Y	Y		
Niemelä, M., Marshall, C. A., Kroll, T., Curran, M., Koerner, S. S., Räsänen, S., & García, F.	2016		Y				
Cooper, V., & Reupert, A.	2017	**Parent:** PSAM; Parenting and MI scale (before & after), Questionnaire, Interviews (n=18)			Y		Y
von Doussa, H., Sundbery, J., Cuff, R., Jones, S., & Goodyear, M.	2017	**Practitioners:** Interviews, Focus groups & Practice enquiry groups	Y	Y			
Solantaus, T.	2017		Y				
Maybery, D. J., Goodyear, M. J., Reupert, A. E., Sheen, J., Cann, W., O'Hanlon, B., & Cuff, R.	2019	**Parents:** Pre post Questionnaire PSS; FAD; Interviews (n=20)	Y	Y	Y		Y
Niemelä, M., Kallunki, H., Jokinen, J., Räsänen, S., Ala-Aho, B., Hakko, H., . . . Solantaus, T.	2019	Population Health data	Y			Y	
Ueno, R., Osada, H., Solantaus, T., Murakoshi, A., & Inoue, T.	2019	**Parents:** BDI- II, Logbook, Questionnaire (safety, self-understanding, mutual fam understanding, parenting, future orientation, wellbeing, treatment motivation & child worries). Pre post measures	Y		Y		
Karibi H. & Arblaster K.	2019	**Practitioners:** Semi-structured interviews	Y	Y			
Allchin, B, Goodyear, M, O'Hanlon, B, Weimand, BM.	2020	**Managers & implementers:** Semi structured interviews	Y				
Allchin, B., O'Hanlon, B., Weimand, B.M., Boyer, F., Cripps, G., Gill, L., Paisley, B., Pietsch, S., Wynne, B., and Goodyear, M.	2020	**Managers, Practitioners & implementers:** Participatory workshops co-constructed, collected and analysed data, (n=5)	Y				
Allchin, B., O'Hanlon, B., Weimand, B.M. and Goodyear, M.	2020	**Practitioners:** Questionnaire	Y	Y			

Author	Year	Measures	implementation outcomes	practitioner outcomes	parent outcomes	child outcomes	family outcomes
Allchin, B., Weimand, B.M., O'Hanlon, B. and Goodyear, M	2020	**Implementers:** Questionnaire and Semi-structured interview	Y				
Giannakopoulos G., Solantaus T., Tzavara C. and Kolaitis G	2021	**Parents:** Questionnaire; BDI-SF; SSAI; SAS-SR adapted; FAD-GF; OSSS-3 (adapted). **Child:** SDQ; CDI; SCARED; KIDSCREEN-27 **4 time periods:** Baseline, 4 10 and 18 month follow up	Y	Y	Y	Y	Y
Nicholson, J.; English, K.; Heyman, M.	2021	**Managers & Practitioners:** Attendance at training, Questionnaire (satisfaction, use, usability), Most significant change story	Y	Y			

Beck depression Inventory (BDI); Beck depression Inventory -II (BDI- II); Beck Depression Index short form (BDI-SF);
Children's Depression Inventory (CDI);
Cognitive Attribution Style (CASQ-R);
Family Assessment Device -General Functioning Subscale (FAD-GF); Family Assessment Device -General Functioning index (FAD),
Family Focused Mental Health Practice Questionnaire (FFMHPQ); Family Focused Workforce Questionnaire -shorter version of FFMHPQ (FFWQ);
Family Intervention Schedule (FIS);
Illness Attitude Scale (IAS);
Oslo 3-Item Social Support Scale (OSSS-3);
Parent Adolescent Communication Scale (PACS),
Parenting Self-Agency Measure (PSAM);
Parenting Stress Scale (PSS);
Screen for Child Anxiety Related Emotional Disorders (SCARED);
Social Adjustment Scale Self-Report (SAS-SR);
Spielberger State Anxiety Inventory (SSAI);
Strengths and Difficulties Questionnaire (SDQ); Child Depression Inventory (CDI);
Symptom checklist 90 (SCL-90);
Sense Of Coherence Scale (SOC)
